# The Complete Genome Sequence and Structure of the Oleaginous *Rhodococcus opacus* Strain PD630 Through Nanopore Technology

**DOI:** 10.3389/fbioe.2021.810571

**Published:** 2022-02-17

**Authors:** Andrea Firrincieli, Beatrice Grigoriev, Hana Dostálová, Martina Cappelletti

**Affiliations:** ^1^ Department of Pharmacy and Biotechnology, University of Bologna, Bologna, Italy; ^2^ Institute of Microbiology of the CAS, Prague, Czechia

**Keywords:** *Rhodococcus opacus* PD630, nanopore sequencing, oleaginous bacteria, complete genome, *Rhodococcus* genomics, *Rhodococcus opacus* plasmids, xenobiotic degradation genes, lipid metabolism genes

## Introduction

*Rhodococcus* bacterial strains are characterized by wide metabolic versatility and extraordinary resistance to environmental stresses (de Carvalho et al., 2014; Orro et al., 2015; Cappelletti et al., 2016, 2019; Pátek et al., 2021). The high versatility and adaptability of *Rhodococcus* strains is partly related with large and complex genomes (up to 10.1 Mbp) including high genetic redundancy and the presence of several circular and linear (mega)plasmids, which harbour peculiar catabolic and biosynthetic genes (Cappelletti et al., 2019). Within *Rhodococcus* genus, *R. opacus* strain PD630 is considered a model oleaginous strain for its ability to produce and accumulate lipids (mostly triacyglycerols, TAGs) using different carbon sources, including low-cost and renewable resources such as lignocellulose (Alvarez et al., 1996; Anthony et al., 2019; Cappelletti et al., 2020; Alvarez et al., 2021; Donini et al., 2021). Notably, under specific growth conditions, this strain is capable of accumulating up to 80% of its cellular dry weight in TAGs (Alvarez and Steinbüchel, 2002); that is a rare feature in the prokaryotic and eukaryotic kingdoms. Multi-omic approaches have been applied to obtain system-level information about metabolic and regulatory pathways involved in these biosynthetic processes. Novel molecular tools for genome editing (CRISPR/Cas9 and recombineering) have been recently developed highlighting the possible utilization of *R. opacus* PD630 as synthetic biology platform for lipids production (Donini et al., 2021; Liang and Yu, 2021).

A first assembly of the *R. opacus* PD630 genome was submitted by the Broad Institute in 2011 (Holder et al., 2011) and included 491 contigs. Later in 2014, a “complete” version of the PD630 genome was submitted by the Institute of Biophysics of the Chinese Academy of Sciences (hereafter IBP_PD630) (Chen et al., 2013) that was recently indicated as “Anomalous assembly” and “contaminated” and therefore deleted from NCBI RefSeq. IBP_PD630 reported the PD630 genome to be composed by one chromosome and nine plasmids (two circular and seven linear plasmids). This result was divergent from the typical number of extrachromosomal elements reported for genomes of this genus, five being the maximum number of extrachromosomal elements described in a single *Rhodococcus* strain (Cappelletti et al., 2019). Despite the assembly-related issues, IBP_PD630 has been used as reference genome in many works involving -omics analyses for the detection of genetic determinants involved in aromatics tolerance and conversion into lipids (DeLorenzo et al., 2018; Kim et al., 2019; Chen et al., 2013; Yoneda et al., 2016; Henson et al., 2018).

In this study, we combined Oxford Nanopore sequencing with Illumina high quality data to solve the architecture of the *R. opacus* PD630 genome and to obtain its whole sequence. Here, in addition to the high-quality complete genome sequence, we demonstrate that this strain does not possess nine plasmids as previously stated, but instead harbours one chromosome and three (one linear and two circular) plasmids. Further, by solving the final structure of *R. opacus* PD630, we found that the large linear plasmid included extrachromosomal genes involved in lipid and xenobiotics’ metabolism. This work provides the correct PD630 genomic framework for the development of genetic engineering strategies to boost the application of this strain as microbial cell factory for lipid production.

## Materials and Methods

### Bacterial Growth and Genomic DNA Extraction

*R. opacus* PD630 (DSM 44 193) was purchased from the Leibniz Institute DSMZ-German Collection of Microorganisms and Cell Cultures (Braunschweig, Germany). For genomic DNA extraction, PD630 was cultivated in 50 ml Luria Bertani (LB) broth at 30°C for 24 h under shaking conditions at 150 rpm. The culture was centrifuged at 4°C for 10 min at 5,000 rpm and the whole cell pellet was subjected to the procedure indicated by Cappelletti et al. (Cappelletti et al., 2011) with slight modifications that involved the utilization of a 10% sodium dodecyl sulfate (SDS)-based lysis buffer to disrupt the cells instead of the mechanical treatment (mediated by bead beater). The genomic DNA was quantified via Qubit dsDNA BR assay kit with the Qubit 4.0 fluorometer (Life Technologies).

### Oxford Nanopore Whole Genome Sequencing

The genomic DNA was fragmented for 10 s via sonication and the integrity of the DNA after fragmentation was checked via electrophoresis gel. The sequencing library was performed with the Oxford Nanopore Ligation Sequencing Kit (SQK-LSK110), according to manufacturer’s instructions. The genomic library was loaded on a FLO-MIN106D (chemistry R9.4.1) flow cell and sequenced with the MinION Mk1C device (Oxford Nanopore Technology, ONT). The sequencing run was performed until 3.5 Gb of bases were obtained that corresponded to a total of 694,680 reads. Base calling of the FAST5 data from MinION was carried out with Guppy GPU 5.0 in high-accuracy mode and with default parameters (chunks per runner 256; chunk size 2000; minimum qscore 7). Evaluation of sequencing quality of the basecalled FAST5 data was performed using PycoQC v2.5.2. Sequencing reads were deposited in the Sequence Read Archive (SRA) SRX12606194.

### Sequencing Data Assembly and Annotation

The genome was assembled using Canu assembler v1.2 (Koren et al., 2017) and the draft assembly was corrected using Illumina short reads [SRX875494 (Janet, 2014)] (Illumina HiSeq 2500) with a single round of Pilon v. 1.24 (Walker et al., 2014). The draft assembly was finally circularized using Circlator (Hunt et al., 2015) indicating the *dnaA* gene of *R. opacus* PD630 (OPAG_07542) as the chromosomal sequence start. A final round of polishing was performed with Berokka to trim overhanging ends from plasmids. Finally, genome completeness was assessed via BUSCO (Manni et al., 2021) and annotated *de novo* using the standalone version of Prokaryotic Genome Annotation Pipeline (PGAP) (Li et al., 2020).

### Comparative Analysis With Other PD630 Genome Assembly Versions

The synteny analysis between the overall organization of PD630 Chen’s assembly (Chen et al., 2013) and the genome presented in this work was performed by Sibelia (Minkin et al., 2013). Liftoff was used to identify the PD630 genes from Chen’s assembly that did not map against our genome due to the presence of miscalled nucleotides, insertion/deletions (indels), and structural variants. A gene was considered to successfully map when the alignment coverage and sequence identity was equal to 100%.

### Functional Annotation of Protein Coding Genes in Plasmids of *R. opacus* Strains

Proteins harbored by plasmids of *R. opacus* strains and *R. jostii* RHA1 were downloaded from RefSeq and annotated using the functional database KEGG with KofamKOALA (Aramaki et al., 2019) (Supplementary Table S1). According to the Genome Taxonomy Database (Chaumeil et al., 2019), we included in the analysis the only *R. opacus* strains with a complete genome currently available in RefSeq (Accessed: December 10th, 2021), i.e., *R. opacus* B4, *R. opacus* DSM 44 186, *R. opacus* 1CP, and *R. opacus* KT112-7.

## Interpretation of Data Set

### Genome Assembly and Annotation

After base calling a total of 667,679 reads with a median Phred score of 13.43, and a median length of 3.1 Kb were generated (Supplementary Figures S1A,B). The draft assembly generated by Canu consisted of four contigs with a total length of 9.16 Mbp. After one polishing round in Pilon using Illumina HiSeq paired-end reads, the size of the Canu-assembled contig slightly increased mostly because of single and/or di-nucleotide insertions (Supplementary File 1). Large variants (>100 bp) were manually checked via blastn. As a result, all of them perfectly matched with sequences of *R. opacus* PD630 (Supplementary File 1). A change in the final size (>10 Kb) of the contigs RoPD630, pRoPD630_2, and pRoPD630_3 was observed after the circularization and trimming steps (Circlator + Berokka) (Table 1), due to the removal of duplicated sequences at their ends. The presence of start-end overlaps in contigs is a well-known behaviour of Canu assembler which tends to generate contigs with a contiguity above 100% (Wick and Holt, 2021).

**TABLE 1:** Length (bp) of chromosome and plasmids throughout the correction and circularization steps.

Predicted topology	RoPD630 (CP080954)	pRoPD630_1 (CP080955)	pRoPD630_2 (CP080956)	pRoPD630_3 (CP080957)
	Circular	Linear	Circular	Circular
Canu	8,411,399	538,757	210,131	121,266
Pilon	8,412,872	538,909	210,169	121,283
Circlator	8,374,353	538,909	210,169	85,051
Berokka	8,374,353	538,909	67,985	85,051
Final bp - Canu bp^a^	−37,046	+152	−42,145	−36,215

a Difference in the number of bp after all the steps with respect to the initial Canu output.

The final assembly (obtained after circularization and trimming) provided a genome with an overall size of 9.16 Mb, including one circular chromosome (RoPD630) of 8.37 Mb and three plasmids, one linear (pRoPD630_1) and two circular (pRoPD630_2 and pRoPD630_3). The average G + C content of the circular chromosome was 67.38% whereas plasmids pRoPD630_1, pRoPD630_2, and pRoPD630_3 showed lower G + C values, i.e., 65.27, 65.06, and 63.91%, respectively (Table 1). The genome completeness was checked via BUSCO, resulting in 100% completeness and 1.4% duplication. According to the PGAP, FABIT_PD630 genome contained 8,280 genes, of which 7,972 protein coding genes, 66 RNA genes, and 242 pseudo-genes.

### Genome Assembly Comparison Between the IBP_PD630 and FABIT_PD630 Genomes

We specifically performed comparative analysis between the IBP_PD630 and FABIT_PD630 genomes to analyse their differences at structural and genetic levels. As a result, FABIT_PD630 possesses a lower number of genes as compared with IBP_PD630, i.e., 8,280 against the 9,005 genes detected in IBP_PD630 assembly. The different number of genes can be attributed to the distinct annotation pipelines that were used in the two works and/or to actual differences in the genome sequence (i.e., sequence variants). To get deep into the reason on this discrepancy, we investigated the possibility that missing genes were due to structural and nucleotide variants. Therefore, we only focused on the identification of the IBP_PD630 genes that did not map with a perfect alignment (i.e., alignment coverage and sequence identity of 100%) with FABIT_PD630 assembly. As a result, only 238 IBP_PD630 genes (around 30% of the number of genes missing in FABIT_PD630 as compared to IBP_PD630) did not align with a perfect match. Furthermore, the number of genes not mapping to our genome was reduced to 84 (<1% of all the genes of Chen’s assembly) when a coverage and sequence identity of 90% was imposed (Supplementary File 2). These results indicate that the differences observed between FABIT_PD630 and IBP_PD630 in terms of identified gene number were mainly due to the annotation pipeline. Annotation pipelines are known to have different sensitivity to misannotations and therefore to the detection of open reading frames (Monnahan et al., 2020).

**FIGURE 1:**
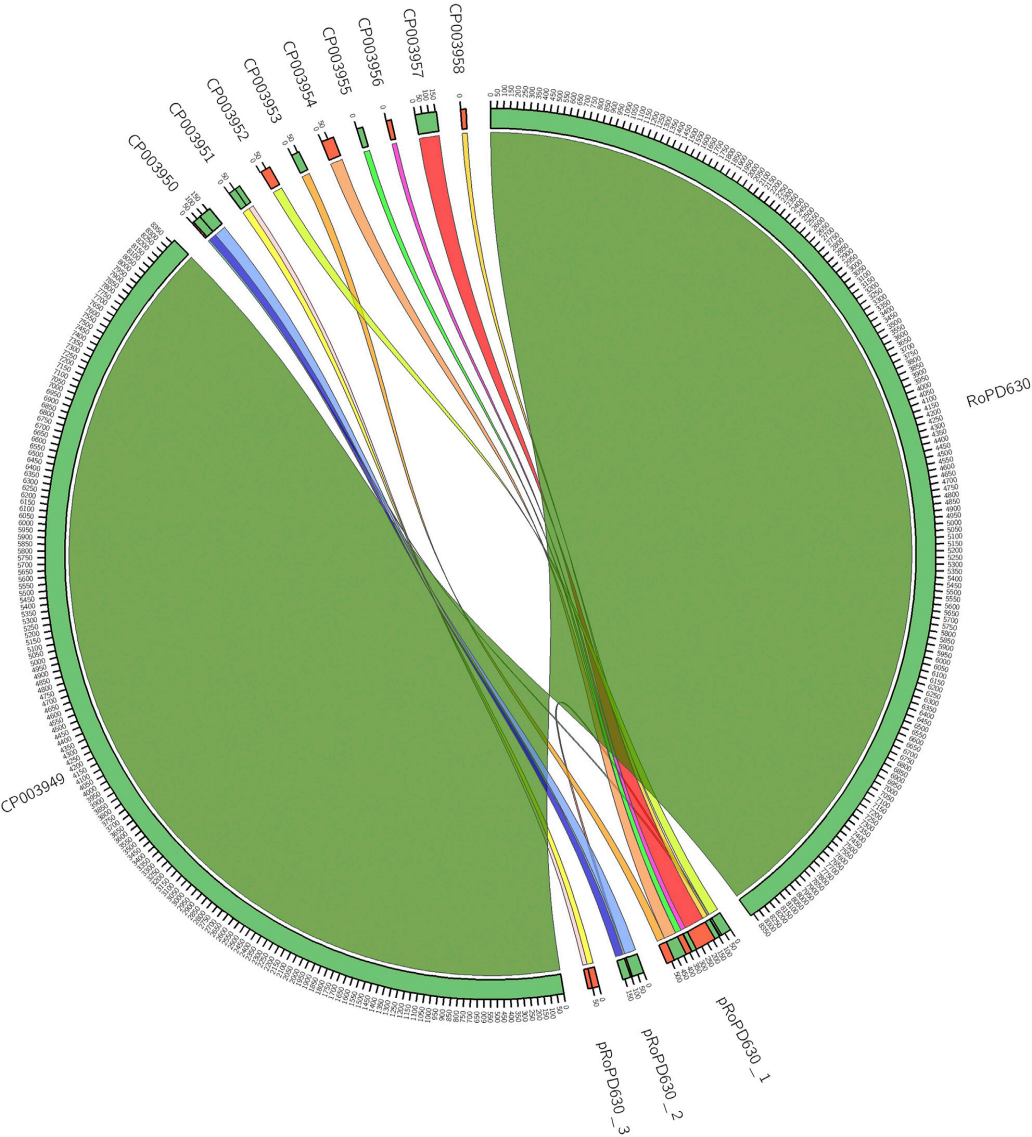
Inferred synteny between IBP_PD630 and FABIT_PD630 assemblies. A minimum synteny block of 10 Kb was selected.

Syntenic analysis of IBP_PD630 plasmids showed near-perfect match between the seven linear plasmids (CP03952-CP03958) of IBP_PD630 and the linear plasmid pRoPD630_1 of FABIT_PD630. This indicates that the sequences CP03952-CP03958 do not correspond to distinct plasmids but represent the fragments of a single linear megaplasmid present in *R. opacus* PD630. Similarly, a nearly-perfect match was observed between each of the circular plasmids CP03950 and CP03951 and the circular plasmids pRoPD630_2 and pRoPD630_3, respectively (Figure 1). Therefore, these results demonstrate that *R. opacus* PD630 possesses only three plasmids instead of nine. The difference between the genetic structure of the two genomes can be attributed to the distinct sequencing and library preparation technologies used in the two works to assemble BIP_PD630 and FABIT_PD630. Indeed, Chen et al. (Chen et al., 2013) carried out a primary assembly using 454 pyrosequencing reads, followed by scaffolding step using Illumina mate-pair library with insert size of 3,000 bp. Although Illumina mate-pair libraries can be successfully used to improve contiguity and therefore close gaps between contigs (Wetzel et al., 2011), the utilization of a single mate-pair library could have been not able to correctly solve repetitive regions of different sizes. Indeed, the capacity of finding links between contigs is limited by the length of the gap itself (Wetzel et al., 2011). An additional downside of Illumina mate-pair reads that might have hampered a correct assembly is the uneven sequencing depth introduced during PCR amplification step which is prone to GC content related bias (Sohn and Nam, 2016). Conversely, long-read sequencing technologies, including the ONT sequencing used in our work, are not typically affected by these issues as they apply library preparation strategies that are PCR-free and therefore less biased towards regions with high AT/GC content. On the other hand, in order to cope with the error rate limits of the ONT technology (Laver et al., 2015), we used high quality Illumina sequencing data (available online under the accession number SRX875494) to correct the possible sequencing errors introduced during long-reads sequence generation.

Our work provides the definitive structure of the plasmids of PD630 that have drawn attention in relation with the aromatics bioconversion capacity of this strain. In this regard, Henson et al. (Henson et al., 2018) showed that Adaptive Laboratory Evolution (ALE) of PD630 strain that was sequentially cultured on different aromatics led to multiple plasmid loss events. The selective loss of extrachromosomal elements seemed to benefit the PD630 growth under specific conditions by leading to the deletion of superfluous genes and saving in large plasmid replication costs (DeLorenzo et al., 2018). Furthermore, the PD630 plasmids were found to harbour several genes encoding enzymes involved in the catabolism of hetero- and poly-cyclic aromatic compounds as well as multiple uncharacterized mono and dioxygenases. This observation pointed out the interest of these plasmids as potential targets for genetic engineering strategies and bacterial strain improvement (Cappelletti et al., 2019; Anthony et al., 2019; DeLorenzo et al., 2018). The knowledge of the correct PD630 genome structure and plasmid organization provided by our work is therefore crucial to assess the molecular and genetic bases of the PD630 peculiar metabolic capacities and to correctly design strain improvement strategies.

### Functional Profiles of the *R. opacus* Species Plasmids

**FIGURE 2:**
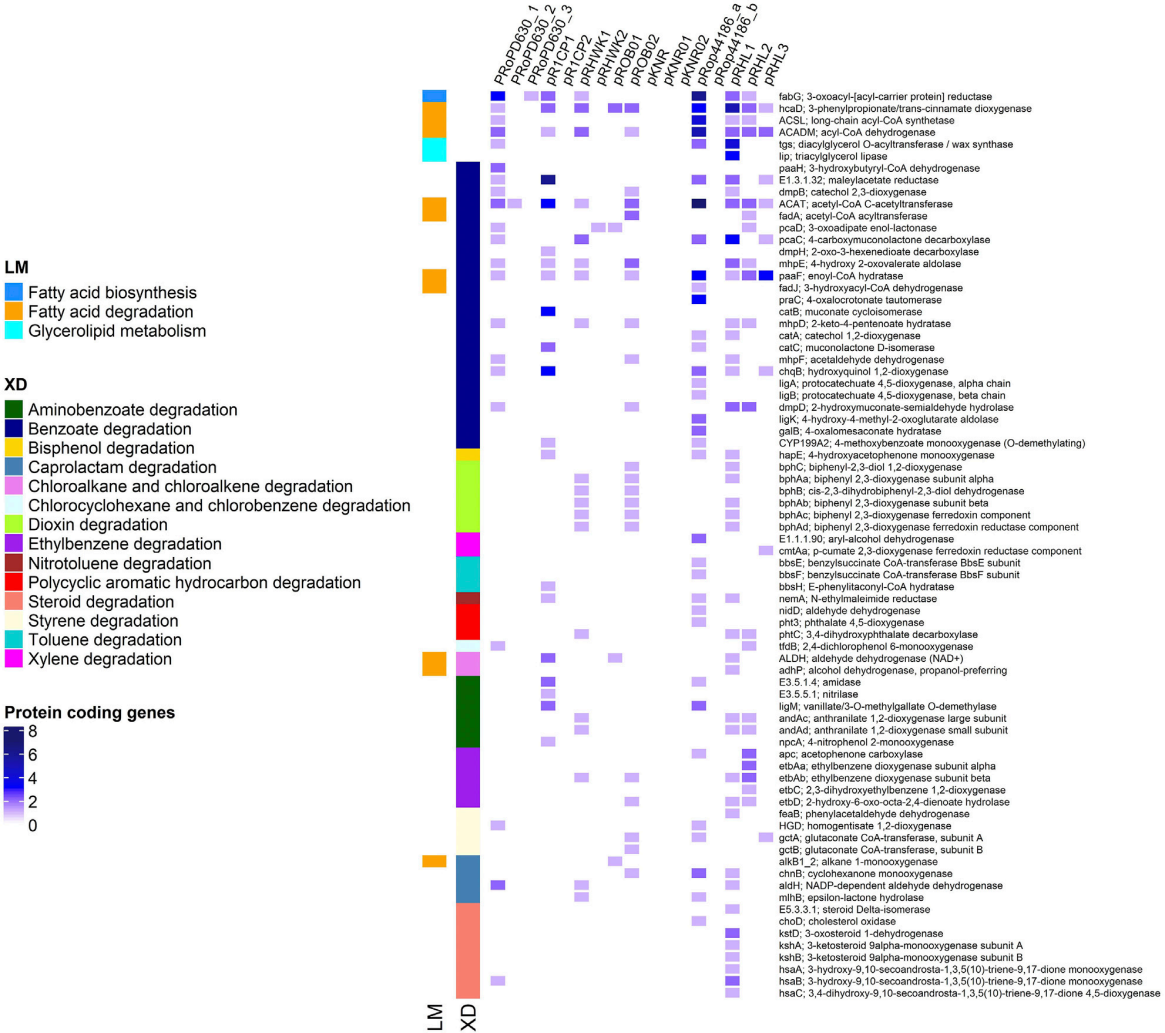
Functional profiling of the protein coding genes involved in xenobiotic degradation (XD) and lipid metabolism (LM) in plasmids of *R. opacus* species and *R. jostii* RHA1 (pRHL). The *Rhodococcus* opacus strains analyzed in this study were *R. opacus* PD630 (pRoPD630), *R. opacus* 1CP (pR1CP), *R. opacus* KT112-7 (pRHWK), *R. opacus* B4 (pROB and pKNR), and *R. opacus* DSM 44 186 (pRop44186).

To gain a better understanding on the repertoire of catabolic potential harbored by each of the three *R. opacus* PD630 plasmids defined in this study, their KEGG functional annotation was performed. Additionally, a comparative analysis of all the plasmids of *R. opacus* strains available in the database (with a complete genome and unanimously identified as belonging to this species) was conducted. *R. jostii* RHA1 was also included in the analysis as the model strain of *Rhodococcus* genus. As a result, the percentage of KEGG orthologues detected in PD630 was 30.8% in pRoPD630_1, 18.7% in pRoPD630_2, and 11.8% in pRoPD630_3. All the three PD630 plasmids harbored genes associated with lipid metabolism, biosynthesis of vitamin, and secondary metabolites, whereas only pRoPD630_1 and pRoPD630_2 included also genes related to carbon, energy, amino acid, and nucleotide metabolism and xenobiotic degradation (Supplementary Table S1). However, pRoPD630_1 included a significantly higher number of genes associated to all of these metabolisms as compared to pRoPD630_2 and pRoPD630_3. In particular, pRoPD630_1 showed a high number of genes associated with xenobiotics degradation and fatty acid catabolism/anabolism that are the metabolic capacities mostly studied for this strain and in general for *R. opacus* species (Anthony et al., 2019; Donini et al., 2021). Conversely, in the PD630 genome version of (Chen et al., 2013), these genes were distributed over the seven scaffolds that were erroneously identified as separate plasmids. These gene functions were also found to be mostly co-localized on one of the several plasmids carried by the other *R. opacus* strains under analysis, while in *R. jostii* RHA1 they were scattered (Figure 2). By resolving the final structure of all the PD630 replicons, we have now the full comprehension of the organization of biotechnologically relevant genes also associated with its plasmids.

## Data Availability

The original contributions presented in the study are publicly available. This data can be found here: https://doi.org/10.6084/m9.figshare.16944811 for the supplementary file 1 and 2; https://www.ncbi.nlm.nih.gov/assembly/GCF_020542785.1 for the genome assembly; https://www.ncbi.nlm.nih.gov/sra/SRX12606194 for the Nanopore sequencing reads.

## Supplementary Material

Supplementary File 1 Pilon polishing round results.

Supplementary File 2 Liftoff analysis results of IBP_PD630 genes mapped over FABIT_PD630 assembly using increasing coverage and identity thresholds: (i) 100% coverage and identity, (ii) 95% coverage and identity, and (iii) 90% coverage and identity. Each folder includes two files, “mapped” and “unmapped.” The “mapped” file is a GFF (General Feature Format) file including all IBP_PD630 genes successfully mapped against FABIT_PD630 and their coordinates. Conversely, the “unmapped” file is a text file reporting the IBP_PD630 genes that do not satisfy the minimum coverage and identity values specified in the Liftoff analysis. GFF files provide the framework to identify the IBP_PD630 genes in the FABIT_PD630 assembly Finally, a Sequence Alignment Map (SAM) file is provided to inspect the alignment of IBP_PD630 genes over FABIT_PD630 genome.
