# Supplementary material for: The Complete Genome Sequence and Structure of the Oleaginous Rhodococcus opacus Strain PD630 Through Nanopore Technology
Source: Front Bioeng Biotechnol. 2022 Feb 17;9:810571. doi: 10.3389/fbioe.2021.810571 (PMC8892189; doi:10.3389/fbioe.2021.810571)
Supplement: Supplementary file 1 [file Image1.PDF]

Supplementary Material

1 SUPPLEMENTARY DATA

1.1 Figures

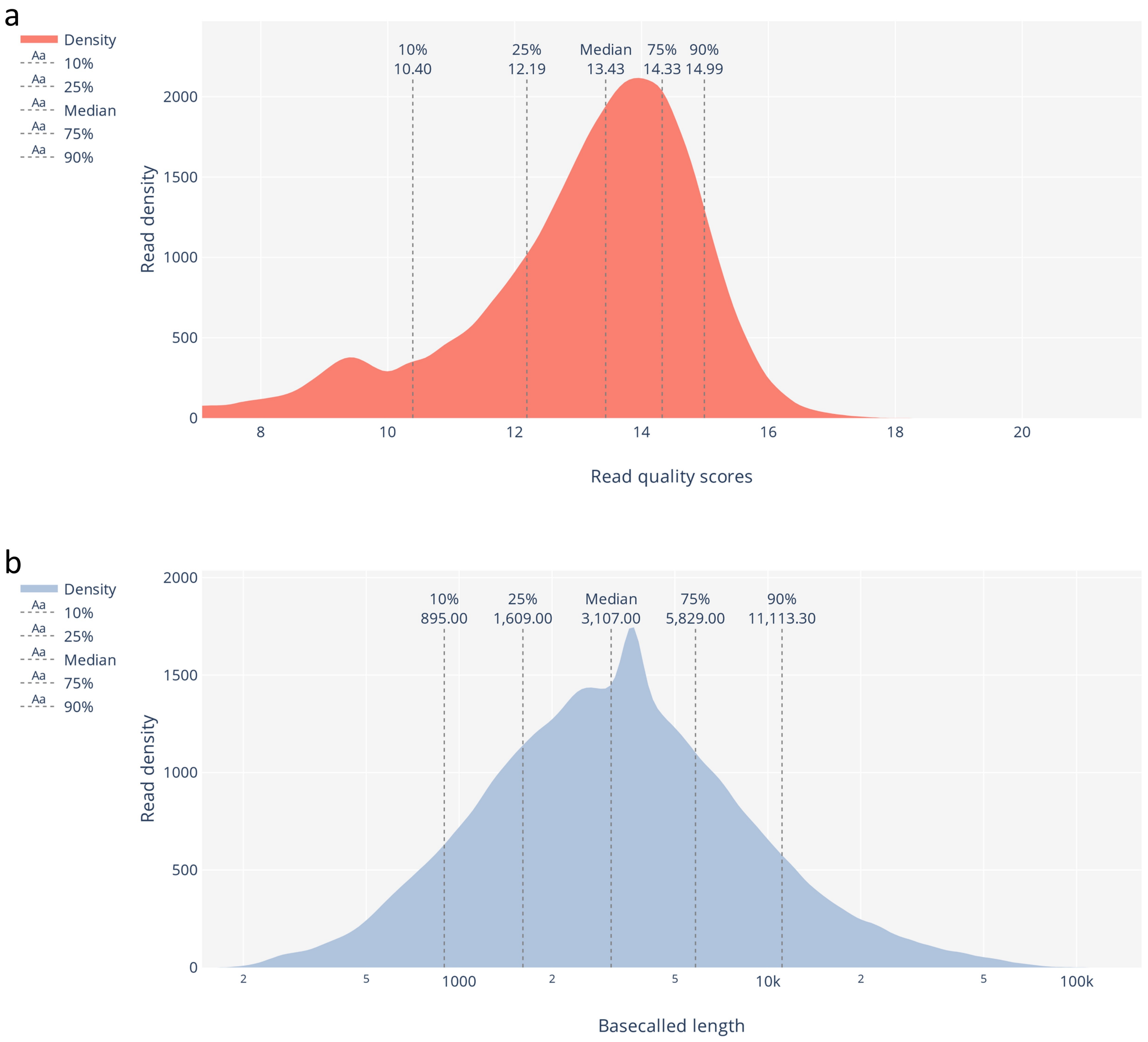

Figure S1. Quality (a) and length (b) distribution of basecalled MinION reads. The quality and length distributions are calculated only for reads with quality score > 7.
